# *PBRM1* mutations might render a subtype of biliary tract cancers sensitive to drugs targeting the DNA damage repair system

**DOI:** 10.1038/s41698-023-00409-5

**Published:** 2023-07-03

**Authors:** Kai Zimmer, Florian Kocher, Gerold Untergasser, Brigitte Kircher, Arno Amann, Yasmine Baca, Joanne Xiu, W. Micheal Korn, Martin D. Berger, Heinz-Josef Lenz, Alberto Puccini, Elisa Fontana, Anthony F. Shields, John L. Marshall, Michael Hall, Wafik S. El-Deiry, David Hsiehchen, Teresa Macarulla, Josep Tabernero, Renate Pichler, Moh’d Khushman, Upender Manne, Emil Lou, Dominik Wolf, Viktorija Sokolova, Simon Schnaiter, Alain G. Zeimet, Pat Gulhati, Gerlig Widmann, Andreas Seeber

**Affiliations:** 1 Department of Hematology and Oncology, Comprehensive Cancer Center Innsbruck (CCCI), Medical University Innsbruck (MUI), Innsbruck, Austria; 2 Tyrolean Cancer Research Institute, Innsbruck, Austria; 3 Caris Life Sciences, Phoenix, AZ USA; 4 Department of Medical Oncology, Inselspital, University of Bern, Bern, Switzerland; 5 Division of Medical Oncology, Norris Comprehensive Cancer Center, Keck School of Medicine, University of Southern California, Los Angeles, CA USA; 6 Medical Oncology Unit 1, Ospedale Policlinico San Martino, Genoa, Italy; 7 Drug Development Unit, Sarah Cannon Research Institute UK, Marylebone, London, UK; 8 Department of Oncology, Karmanos Cancer Institute, Wayne State University, Detroit, MI USA; 9 Ruesch Center for The Cure of Gastrointestinal Cancers, Lombardi Comprehensive Cancer Center, Georgetown University Medical Center, Washington, DC USA; 10 Department of Hematology and Oncology, Fox Chase Cancer Center, Temple University Health System, Philadelphia, PA USA; 11 Department of Pathology and Laboratory Medicine, Cancer Center at Brown University, Providence, RI USA; 12 Division of Hematology and Oncology, Department of Internal Medicine, University of Texas Southwestern Medical Center, Dallas, TX USA; 13 Medical Oncology Department, Vall d’Hebron Hospital Campus and Institute of Oncology (VHIO), IOB-Quiron, Barcelona, Spain; 14 Department of Urology, Comprehensive Cancer Center Innsbruck, Medical University of Innsbruck, Innsbruck, Austria; 15 O’Neal Comprehensive Cancer Center, the University of Alabama at Birmingham, Birmingham, Al USA; 16 Masonic Cancer Center, University of Minnesota, Minneapolis, MN USA; 17 Department of Nuclear Medicine, Provincial Hospital of Bolzano (SABES-ASDAA), Teaching Hospital of the Paracelsus Medical Private University, Bolzano-Bozen, Italy; 18 Institute of Human Genetics, Medical University of Innsbruck, Innsbruck, Austria; 19 Department of Obstetrics and Gynaecology, Comprehensive Cancer Center Innsbruck, Medical University of Innsbruck, Innsbruck, Austria; 20 Rutgers Cancer Institute of New Jersey, New Brunswick, NJ USA; 21 Department of Radiology, Medical University of Innsbruck, Innsbruck, Austria

**Keywords:** Oncology, Cancer genomics

## Abstract

*Polybromo-1* (*PBRM1*) loss of function mutations are present in a fraction of biliary tract cancers (BTCs). *PBRM1*, a subunit of the PBAF chromatin-remodeling complex, is involved in DNA damage repair. Herein, we aimed to decipher the molecular landscape of *PBRM1* mutated (mut) BTCs and to define potential translational aspects. Totally, 1848 BTC samples were analyzed using next-generation DNA-sequencing and immunohistochemistry (Caris Life Sciences, Phoenix, AZ). siRNA-mediated knockdown of *PBRM1* was performed in the BTC cell line EGI1 to assess the therapeutic vulnerabilities of ATR and PARP inhibitors in vitro. *PBRM1* mutations were identified in 8.1% (*n* = 150) of BTCs and were more prevalent in intrahepatic BTCs (9.9%) compared to gallbladder cancers (6.0%) or extrahepatic BTCs (4.5%). Higher rates of co-mutations in chromatin-remodeling genes (e.g., *ARID1A* 31% vs. 16%) and DNA damage repair genes (e.g., *ATRX* 4.4% vs. 0.3%) were detected in *PBRM1*-mutated (mut) vs. *PBRM1*-wildtype (wt) BTCs. No difference in real-world overall survival was observed between *PBRM1*-mut and *PBRM1*-wt patients (HR 1.043, 95% CI 0.821–1.325, *p* = 0.731). In vitro, experiments suggested that PARP ± ATR inhibitors induce synthetic lethality in the *PBRM1* knockdown BTC model. Our findings served as the scientific rationale for PARP inhibition in a heavily pretreated *PBRM1-*mut BTC patient, which induced disease control. This study represents the largest and most extensive molecular profiling study of *PBRM1-*mut BTCs, which in vitro sensitizes to DNA damage repair inhibiting compounds. Our findings might serve as a rationale for future testing of PARP/ATR inhibitors in *PBRM1-*mut BTCs.

## Introduction

The prognosis of advanced/metastatic biliary tract cancers (BTCs) remains poor^1^, despite approval of novel therapies targeting *FGFR2* gene fusions and *IDH* mutations^2,3^. Unfortunately, only a minor fraction of BTC patients are eligible for these specific treatments. In recent years the chromatin remodeling system has gained increased attention as a potential therapeutic target in several tumor types. Chromatin remodeling is a tightly regulated process controlling gene accessibility for transcription and thus regulating gene expression^4,5^. The Switch/Sucrose Non-Fermentable (SWI/SNF) chromatin remodeling complex represents a key component of chromatin remodeling^6^. Dysregulation of SWI/SNF due to loss of function mutations in genes—such as *ARID1A*, *SMARCB1*, and Polybromo-1 (*PBRM1;* also known as *BAF180*)—is present in up to 20% of malignancies^7^, but, to date, no targeted treatment related to the chromatin remodeling process has been approved.

In BTCs, *PBRM1* mutations are present in 5–21% of cases^8,9^. In addition to chromatin remodeling, *PBRM1* further participates in the repair of DNA double-strand breaks (DSB) via ATM phosphorylation^10^. Most genetic alterations of *PBRM1* induce loss of function, consequently impairing DNA damage repair. Thus, *PBRM1-*mutated (mut) BTCs might be sensitive to agents targeting DNA damage repair. Indeed, the synthetic lethal effect of poly-(ADP-Ribose) polymerase (PARP) inhibitors (PARPi) has been suggested in an in vitro renal cell carcinoma model harboring *PBRM1* mutations^11^. PARPi induces synthetic lethality in cells lacking the ability to repair DSBs, which was first demonstrated in Breast Cancer Gene 1 or 2 (*BRCA1* or *BRCA2*)-mut tumors. Thus, PARPi has been approved for the treatment of *BRCA*-mut breast^12–14^, ovarian^15–17^, pancreatic^18^, and prostate cancer^19^. PARPi are currently under clinical investigation in several malignancies harboring mutations in genes involved in DNA damage repairs, such as *RAD51*^20,21^, *PALB2*^22^*, or ARID1A*^23,24^. Moreover, combinations of PARPi with inhibitors of the DNA-damage repair protein ATR (Ataxia Telangiectasia and Rad3 related) are currently under investigation to overcome PARPi resistance^25^.

To the best of our knowledge, the genomic context of *PBRM1* mutations in BTCs has not been investigated so far. Therefore, we aimed to characterize the molecular landscape of *PBRM1*-mut BTCs and investigate the potential therapeutic role of PARP/ATRi in *PBRM1*-mut BTCs.

## Results

### Patient characteristics

**Fig. 1:**
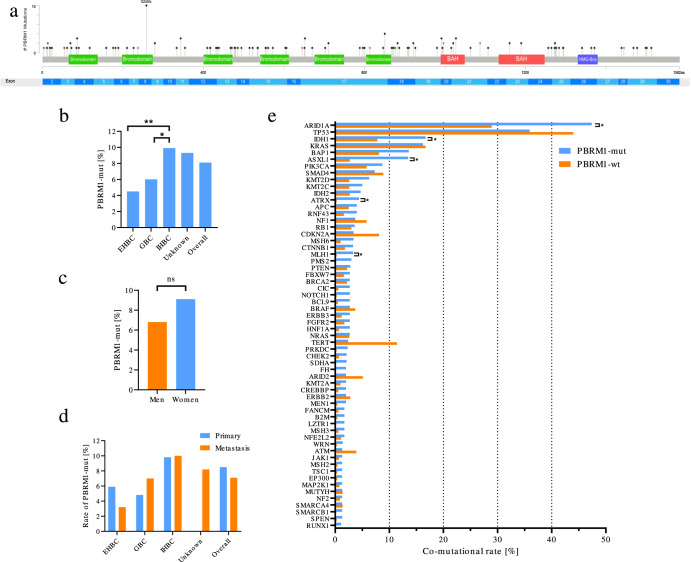
Genomic context of PBRM1-mutated BTCs reveals high co-occurrence of mutations in chromatin remodeling genes. **a** Lollipop plot showing the distribution of the detected *PBRM1*-mutations. The N258fs-mutation (Exon 8) was the most frequent pathogenic variant detected in our cohort. **b** Means of *PBRM1* mutations according to anatomic location (extrahepatic biliary tract cancer (EHBC), gallbladder cancer (GBC), and intrahepatic biliary tract cancer (IHBC). Statistically significant differences by two-sided Man–Whitney U are indicated as *p < 0.05, **p < 0.01. **c** Sex-specific rates of *PBRM1* mutations. Chi-Square test not significant (ns). **d** Rates of *PBRM1* mutations by anatomic location stratified by sampling location (Primary vs. Metastasis). **e** Bar plot showing the rate of co-mutations of the indicated genes in *PBRM1*-mut samples compared to *PBRM1*-wt samples. Statistically significant differences by two-sided Man–Whitney *U* test after correction for multiple testing using are indicated as *q < 0.05. **f** Bar plot showing the rate of co-mutation stratified by anatomic location. Stars indicate significance level (chi-square test). *p < 0.05, **p < 0.01,***p < 0.001, all other not significant. IHBC intrahepatic biliary tract cancer, EHBC extrahepatic biliary tract cancer, GBC gallbladder cancer.

In total, 1848 BTC samples were centrally analyzed. Of these, 67.5% (*n* = 1249) were derived from primary tumor sites and 32.5% from metastatic lesions (*n* = 593). Altogether, 232 samples harbored different variants within the *PBRM1* gene and 150 (8.1%) of these alterations were classified as pathogenic (frameshift: 51.4%; nonsense: 32.0%; splicing: 16.6%). The most frequently detected *PBRM1* point mutation was the p.N258fs variant (*n* = 10, 6.6%; c.773delA (*n* = 3) and c.773dupA (*n* = 7)) followed by the p.R850* variant (*n* = 4, 2.6%; all c.2548 C > T; Fig. 1a). All pathogenic/likely pathogenic variants identified in the cohort are provided in Supplementary Table 1.

*PBRM1* mutation frequencies were detected at the highest frequencies in intrahepatic BTC (9.9%, *n* = 103/1045; IHBCs) followed by gallbladder cancer (6.0%, *n* = 29/484) and extrahepatic BTC (4.5%, *n* = 11/244, EHBCs, Fig. 1b). The median variant allele frequency (VAF) of PBRM1-mutations was 31% (range 6–74%) and did not differ significantly between anatomical subsites (IHBC: 31%, EHBC 19%, GBC 31%, unclear location 43%, Supplementary Fig. 2). There was no difference in *PBRM1* mutation frequencies between women and men (9.1% vs. 6.8%, *p* = 0.0856, Fig. 1c). Frequencies of *PBRM1-*mut tumors were comparable between samples derived from primary location vs. metastatic lesions (8.5% vs. 7.1%, *p* = 0.3, Fig. 1d). Patients with *PBRM1-mut* BTCs were older relative to *PBRM1-*wildtype (wt) patients (median age: 69 vs. 66 years, *p* = 0.0416) (Table 1).

**Table 1:** Patient characteristics.

		*PBRM1*-mut *n* (%)	*PBRM1*-wt *n* (%)	*p*-value
Age (median)		69	66	0.0416^a^
Sex	Men	55 (6.8)	750 (93.2)	0.0856^b^
	Women	95 (9.1)	948 (90.9)	
Anatomical location	EHBC	11 (4.5)	233 (95.5)	0.014^b^
	GBC	29 (6.0)	455 (94.0)	
	IHBC	103 (9.9)	942 (90.1)	
	Unknown	7 (9.3)	68 (90.7)	
Sampling location	Primary	106 (8.5)	1143 (91.5)	0.300^b^
	Metastasis	42 (7.1)	551 (92.9)	

*EHBC* extrahepatic biliary tract cancer, *GBC* gallbladder cancer, *IHBC* intrahepatic biliary tract cancer.

^a^Two-sided Mann–Whitney *U* test.

^b^Chi-square test.

### *PBRM1*-mutated BTCs differ in key driver mutations compared to *PBRM1*-wildtype samples

The most frequent concomitant molecular alterations detected in *PBRM1*-mut BTCs were mutations in *ARID1A* (47.4%), *TP53* (35.9%), *IDH1* (16.7%), *KRAS* (16.2%), *BAP1* (13.6%), and *ASXL1* (13.5%) (Fig. 1e).

Regarding the rate of co-mutations, there were significant differences between *PBRM1*-mut and *PBRM1*-wt BTCs for *ARID1A* (47.4% vs. 28.9%, *q* = 0.015), *IDH1* (16.7% vs. 7.8%, *q* = 0.01551), *ASXL1* (13.5% vs. 2.7%, *q* = 0.015), *ATRX* (4.4% vs. 0.3%, *q* = 0.015) and *MLH1* (3.3% vs. 0.3%, *q* = 0.029). No differences were evident for frequently observed alterations in BTC (26), such as mutations in *TP53* (35.9% vs. 44.0%, *q* = 0.735) and *KRAS* (16.2% vs. 16.6%, *q* = 1).

Generally, in comparison to *PBRM1*-wt cases, there were lower rates of copy number alterations (CNA) in the *PBRM1*-mut cohort. Most frequent CNAs were present in *FGFR3* (2.8%), *ERBB2* (2.8%), and *FGF3* (1.5%), compared to 1.8%, 4.4%, and 2.4% in *PBRM1-*wt samples, respectively (no significant differences; Supplementary Fig. 3).

In the overall cohort, we identified a higher rate of MSI-H in the *PBRM1*-mut cohort compared to *PBRM1*-wt (8.7% vs. 2.1%, *p* < 0.001). Moreover, *PBRM1-*mut samples were characterized by a higher median TMB (median TMB: 4 mut/Mb vs. 3 mut/Mb, *p* < 0.0001). The rate for patients with a TMB ≥ 10 mut/Mb was 4.0% in *PBRM1-*wt vs. 10.1% in *PBRM1-*mut samples (*q* = 0.029). However, when correcting for MSI-H, the difference in the higher rate of TMB was lost (1.5% vs. 2.2%, *p* = n.s., Supplementary Fig. 4).

### The co-mutational landscape varies between anatomical sites

In the next step, we stratified the cohort according to the anatomical sites (IHBC, EHBC, and GBC). Significant differences in co-mutational rates in *TP53* (64% EHBC, 62% GBC, 24% IHBC, *p* < 0.001), *SMAD4* (18% EHBC, 21% GBC, 2% IHBC, *p* < 0.001) or *IDH1* (18% EHBC, 0% GBC, 22% IHBC, *p* = 0.02) were observed. No significant differences were identified for *ARID1A* (43% EHBC, 45% GBC, 50% IHBC, *p* = 0.9) or ATRX (0% EHBC, 8% GBC, 4% IHBC, *p* = 0.6) mutations, nor in MSI-H/dMMR (18% EHBC, 14% GBC, 7% IHBC, *p* = 0.3). PD-L1 IHC expression status (18% EHBC, 11% GBC, 10% IHBC, *p* = 0.7) or LOH > 16% (0% EHBC, 7% GBC, 8% IHBC, *p* = 0.8) status (Fig. 1f). Moreover, higher median TMB levels for EHBC (4 mut/mb) and GBC (4 mut/mb) compared to IHBC (3mut/mb; Supplementary Fig. 5) were noticed.

### Exploratory real-world overall survival

**Fig. 2:**
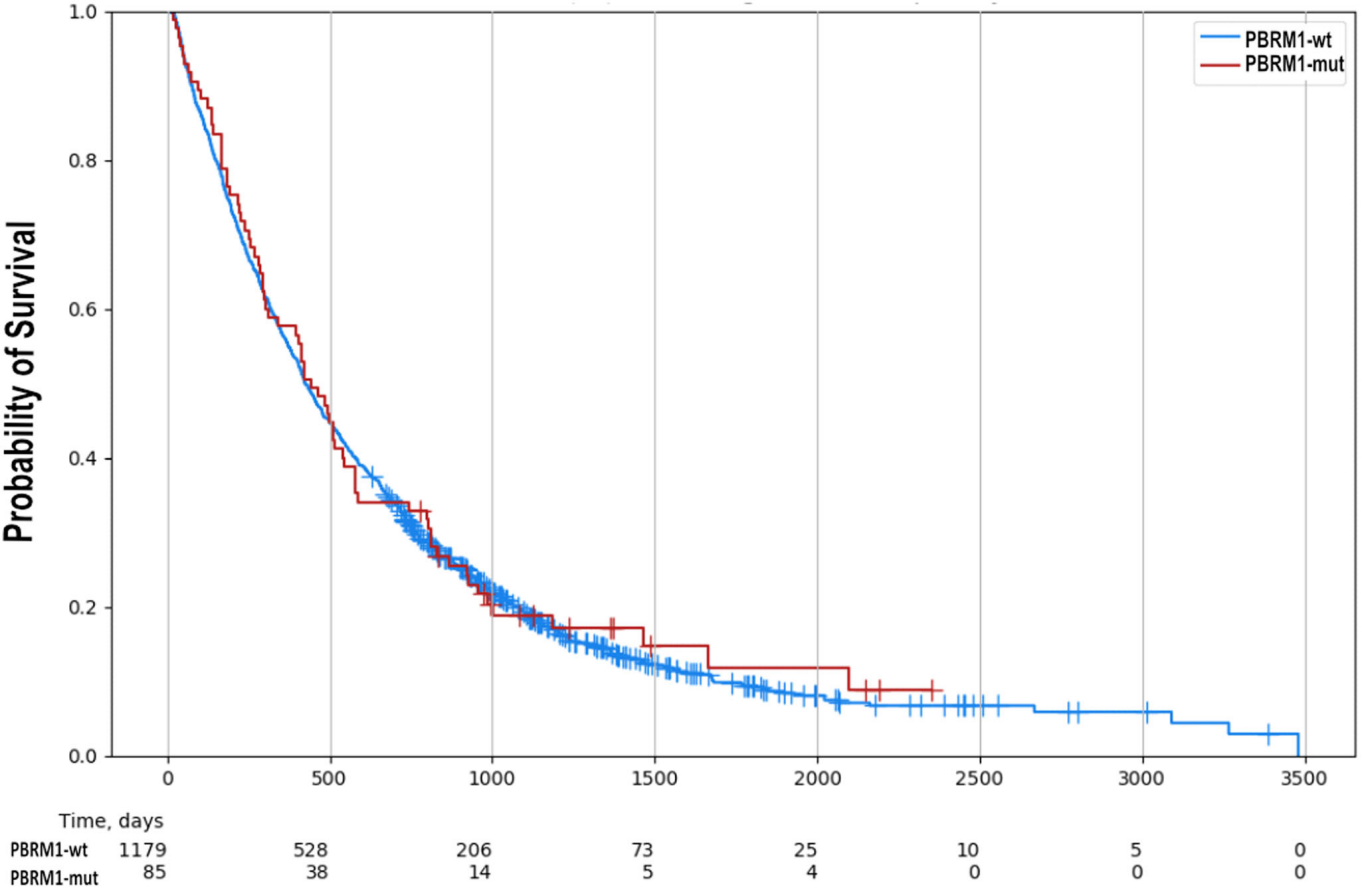
No difference in exploratory real-world survival between PBRM1-mut and PBRM1-wt tumors. Univariable analysis revealed no difference in survival between *PBRM1*-mut and PBRM1-wt tumors (median days-to-event for PBRM1-wt: 424 days, PBRM1-mut: 442 days (HR 1.043, 95% CI 0.821–1.325, *p* = 0.731).

In total, 1264 patients with samples from primary tumors were available for exploratory real-world analysis. No difference between *PBRM1*-mut and *PBRM1*-wt patients (median days-to-event for *PBRM1*-wt: 424 days, for *PBRM1*-mut: 442 days; HR 1.043, 95% CI 0.825–1.325, *p* = 0.731) was observed (Fig. 2). Subgroup analysis according to the anatomic location again revealed no difference between *PBRM1*-mut and *PBRM1*-wt patients (IHBC: median days-to-event for *PBRM1*-wt: 388 days, for *PBRM1*-mut: 451 days, HR 1.107, 95% CI 0.891–1.376, *p* = 0.358; EHBC: median days-to-event for *PBRM1*-wt: 495 days, for *PBRM1*-mut: 492 days, HR 0.909, 95% CI 0.556–1.484, *p* = 0.7) (Supplementary Fig. 6).

### Synthetic lethal effect of PARPi and/or ATRi in a *PBRM1*-depleted BTC model

**Fig. 3:**
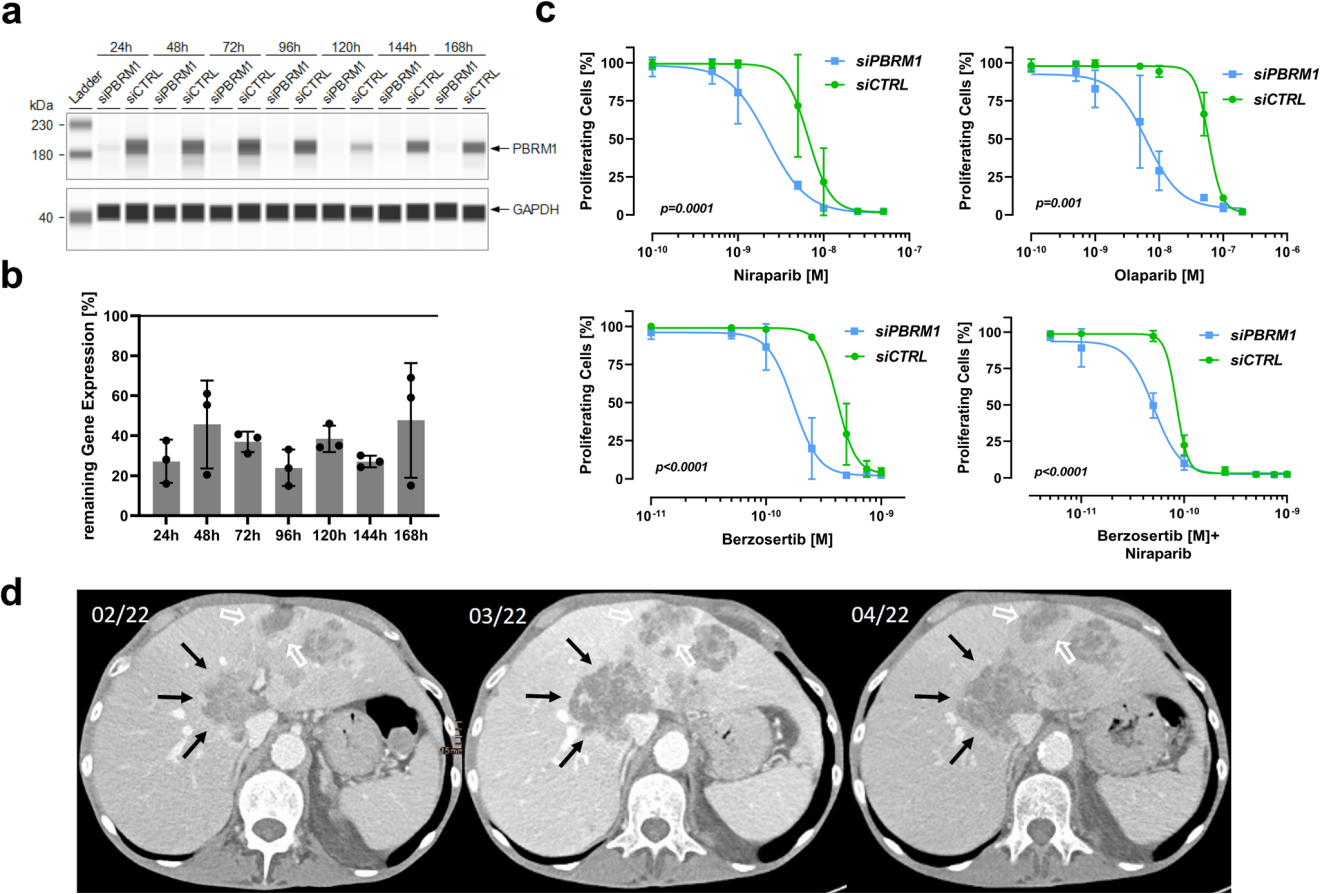
Vulnerability of *PBRM1* deficient biliary tract cancer to DNA damage repair targeting agents. **a** Immunoblot showing the duration of a siRNA-mediated knockdown of *PBRM1* in the EGI-cell line. GAPDH was used as a loading control. A representative of three biological replicates is shown. **b** Relative mRNA expression (mean ± SD of at least three independent experiments) of *PBRM1* over 168 h after the siRNA-mediated knockdown. **c** Four parameter least-squares non-linear regression models of dose-response relation comparing treatment with niraparib, olaparib, berzosertib, or niraparib + berzosertib in *PBRM1-*deficient cells to *PBRM1-*proficient cells. Comparison of LogIC50 values of three independent experiments. Points and error bars represent mean ± SD. **d** Abdominal CT-scan of a patient with a *PBRM1*-mut BTC treated with niraparib over an 8-week time course (left: before treatment, middle: 4 weeks after treatment start, right: 8 weeks after treatment start).

Preclinical evidence suggests that, in renal cell carcinoma, *PBRM1* mutations are a synthetic lethal target of PARPi and ATRi^11^. To elaborate the therapeutic vulnerability to the respective target in BTCs, we performed siRNA-mediated knockdown of *PBRM1* in the BTC cell lines EGI1 and CCC5 (results provided as Supplementary Fig. 1). Pathogenic *PBRM1* gene alterations of EGI1 and CCC5 were excluded by whole-exome sequencing (pathogenic driver mutations are displayed in Supplementary Table 2). RT-qPCR and immunoblotting were used to control for the duration and the depth of the *PBRM1-*knockdown on protein (Fig. 3a) and mRNA (Fig. 3b) levels, respectively. Our experiments revealed that *PBRM1*-knockdown led to a reduction in IC_50_ values for PARPi therapy compared to *PBRM1*-proficient cells: niraparib (2.25 µM vs. 6.624 µM, *p* = 0.0001) and olaparib (59.32 µM vs. 70.75 µM, *p* = 0.01). Similar effects of the *PBRM1*-knockdown were observed when treating the cells with the ATRi berzosertib (0.172 µM vs. 0.415 µM, *p* < 0.0001) and the combination of berzosertib and niraparib (0.05 µM vs. 0.08 µM, *p* < 0.0001) (Fig. 3c).

### Clinical translation: treatment of a *PBRM1-*mut BTC patient with niraparib

Our observations, extracted from genomic analyses and in vitro experiments, served as the rationale to use the PARPi niraparib for a heavily pretreated BTC patient harboring a *PBRM1* mutation. A 50-year-old male patient, who had already received three lines of systemic therapies was re-evaluated upon progression at our cancer center. During the disease course, the patient had never achieved stable disease or response upon standard-of-care systemic treatments. Due to the lack of further standard therapeutic options, a re-biopsy of a newly detected hepatic lesion was performed to evaluate potential therapeutic options through molecular profiling by NGS. The profiling revealed a microsatellite-stable tumor with a TMB of 4 mut/Mb and a LOH score of 6%. NGS identified pathogenic variants in *PBRM1* (p.Y998fs, c.2993_3008del16, Exon 20, VAF 16%, NM_018313.4) and in *ARID1A* (p.P1554fs, c.4661delC, Exon 18, VAF 15%, NM_006015.5). No other findings relevant to treatment selection (i.e., no fusion in *FGFR2* and no mutation in *IDH1*) could be identified. The case was discussed within the local molecular tumor board and after informed consent was obtained, PARPi treatment with niraparib was initiated (off-label use). We performed clinical and radiographic follow-ups after 4 and 8 weeks from treatment initiation. During the course of the treatment, the patient did not report any adverse reactions and reported subjectively improved quality of life. The radiographic analysis according to RECIST v1.1 after 4 and 8 weeks was performed on two targets and three non-target lesions and was summarized as a stable disease after four (overall +4.6% lesion size compared to baseline) and eight weeks (+6.2% compared to baseline). We observed a heterogeneous dynamic response in different lesions (Fig. 3d, Supplemental Table S3, Supplemental Fig. S7). Of note, target lesion 2 (hepatic segment IVa), which was used for molecular profiling and in which we identified the above-mentioned alterations, seemed to be stable in size and showed even slight regression over treatment course (−3% after four weeks). Despite disease stabilization, treatment with niraparib was terminated after a major bleeding event, which was not related to the BTC or niraparib, leading to ICU admission approximately 10 weeks after niraparib initiation, impeding further follow-up.

## Discussion

In this study, we present the genomic context of *PBRM1*-mutated BTCs in a large real-world cohort of patients. *PBRM1* mutations were detected in 8.1% of BTC samples, which is comparable with previous studies^8,9^. Our thorough sequencing approach revealed distinct genomic characteristics of *PBRM1-*mut BTCs. The genomic profile of *PBRM1-*mut BTCs is characterized by a high rate of co-mutations in genes involved in chromatin remodelings, such as *ARID1A, ASXL1*, and *ATRX*.

*ARID1A* is a tumor suppressor and represents the most frequently altered gene of the SWI/SNF complex in human cancers^26^. In BTCs, *ARID1A* represents one of the most frequent mutations present in about 20% of cases^27^, and according to our analysis, *ARID1A* mutations were observed in nearly half of the patients (47.4%) with *PBRM1* mutations. In general, chromatin remodeling dysregulations are frequently observed in BTCs and previous data also suggested that mutations in the respective genes were not mutually exclusive^28^. Considering the co-occurrence of *PBRM1* mutations and additional loss of function alterations in chromatin remodeling genes, our findings suggest that the *PBRM1*-mut subgroup is characterized by an impaired chromatin remodeling system.

Beyond gene expression regulation, *PBRM1* has additional properties that are crucial for genomic integrity and stability^4,29^. Given the variety of functions and the complex molecular structure of the SWI/SNF complex, various targeted therapeutic approaches have been investigated. Both *PBRM1* and *ARID1A* mutations are suggested to be synthetic lethal targets for PARPi, raising the question of possible prognostic and predictive value in BTCs.

Recently, it was reported that *PBRM1-mut* renal cell carcinoma shows sensitivity to PARPi^11^. Our in vitro experiments, involving the BTC cell line EGI1 with a siRNA-induced *PBRM1* depletion, revealed sensitization towards PARPi and ATRi. Additionally, the combination of PARPi and ATRi induced a synergistic effect in our *PBRM1*-knockdown BTC cell line model. The combination of PARPi and ATRi is currently under clinical investigation, aiming to overcome PARPi resistance in *BRCA-*deficient cancers^25,30^.

Regarding prognosis, *PBRM1* mutations in BTC were associated with an improved outcome using the TCGA cohort^31^. Analysis using another cohort of resected BTCs, the subgroup of patients harboring mutations in *IDH1*/2, *BAP1*, or *PBRM1* were characterized by better prognosis compared to patients with *TP53/KRAS* mutations^32^. In contradiction to these findings, in our study, exploratory real-world overall survival revealed no prognostic effect of *PBRM1* mutations in BTC.

We reported a case of a heavily pretreated patient with a *PBRM1-*mut BTC who received the PARPi niraparib. Radiographic evidence showed stable disease after four and eight weeks and the patient reported no signs of treatment-related toxicity and subjective well-being, suggesting a clinical benefit. Due to a major bleeding event that was not related to the BTC, but lead to the termination of niraparib treatment, the follow-up time with approximately two months and thus too short to conclude the objective clinical efficacy of the treatment. However, the dynamics of response in different lesions suggest intra-tumor and inter-lesion heterogeneity with respect to the mutational profile. We found the genetic alterations in *PBRM1* and *ARID1A* to be present in 15–16% of tumor cells. The impact of VAF on target mutations as a biomarker of treatment response has not been investigated so far. A meta-analysis (8 studies, *n* = 200), comparing the PARPi response in patients with somatic versus germline *BRCA* mutations, found no difference in treatment response to PARPi treatment in solid tumors^33^. Contradictory to this finding, a small single-center study (*n* = 41) found a higher VAF of somatic *BRCA1/2* mutations to be associated with longer PFS in ovarian cancer patients^34^. The impact of the VAF of *PBRM1* mutations on prognosis and therapeutic outcome is unknown and warrants further investigation. Interestingly, the target lesion which was used for mutational profiling showed stable size with slight regression during the treatment. As we did not obtain material for NGS from other lesions, we can not report on their mutational profile and if the same mutations were present. For our case report, we can speculate, that the *PBRM1* mutation was only present in a subfraction of the tumor mass, and thus not all cells were equally susceptible to PARPi. This relative treatment resistance may be overcome by combinational treatment approaches (e.g., using immune-checkpoint inhibitors or ATRi). However, there is no approved combination currently available, and thus we refrained from combining niraparib with any additional treatment. Still, our in vitro data suggests a synergistic effect of the ATRi berzosertib in addition to PARPi, and this combination is suggested to overcome PARPi resistance^25,30^. Further, it is suggested that PARPi treatment enhances the efficacy of immune-checkpoint inhibitors via neoantigen formation and by activating STING signaling^34,35^.

In our cohort we observed a higher rate of MSI-H and elevated TMB in *PBRM1*-mut patients, suggesting a susceptibility to immune-checkpoint inhibitor treatment, such as pembrolizumab, as it has been approved for tissue agnostic use in MSI-H cancers^36,37^. While MSI-H has been established as a prognostic biomarker for pembrolizumab treatment even for BTC^36^, there are some contradictory findings regarding the *PBRM1*-status. For lung adenocarcinoma and for clear cell renal cell carcinoma, *PBRM1* mutational status was found to be a negative prognostic marker, while for colon adenocarcinoma, gastric cancer, and uterine corpus endometrial carcinoma, *PBRM1* mutations were found to be a positive predictive marker for immunotherapy^38^. However, prospective studies are lacking and there is no current evidence for BTCs. Interestingly, several phase 3 trials are currently ongoing investigating the combination of immune-checkpoint inhibitors and PARPi^39^. For this reason, in future studies, *PBRM1* may be investigated as a prognostic biomarker also for immunotherapy combinations.

Some limitations apply to our study. Due to the retrospective study design and tissue referral from multiple cancer centers, only a fraction of clinical information is available, thus prohibiting further correlation with clinical data. Therefore, we were only able to provide a univariate analysis of exploratory real-world overall survival. Despite survival analysis was performed in a large cohort of patients, we were not able to draw conclusions on the prognostic impact of *PBRM1* mutations. Future studies, accounting for established risk factors are needed to establish the prognostic role of *PBRM1* mutations in BTC. Nevertheless, we are confident, that the large sample size of 1848 patients provides a reliable resolution of the genomic context of *PBRM1-*mut BTCs. The additional results coming from our in vitro model are preliminary and further more detailed mechanistic and efficacy studies are warranted. Also, to gain a deeper understanding of the interplay between *PBRM1* mutations and co-mutations observed in our study additional in vitro and in vivo studies are required. Finally, our case report of a single patient is quite limited as we could not follow up with the patient for longer than 10 weeks.

Herein, we provide evidence that *PBRM1-*mut BTCs are characterized by a distinct molecular profile defined by higher rates of concomitant mutations in further chromatin remodeling genes, which might predict sensitivity to PARPi. In vitro, experiments support our hypothesis. Collectively, our results might serve as a rationale to assess targeted treatment approaches in patients with BTC harboring *PBRM1* mutations.

## Methods

### Patient characteristics

BTC specimens were submitted to Caris Life Sciences and analyses were performed as previously described^40^. In brief, samples of BTC were retrospectively identified in a commercial database between 2012 and 2020. Hematoxylin and Eosin (H&E) slides were prepared for each tumor sample and were reviewed by board-certified pathologists to confirm the diagnosis of BTC. Tumors with a histological diagnosis that was not concordant with the diagnosis of BTC were excluded from this analysis. For each specimen, gene sequencing, amplification, and protein expression data were evaluated. Besides the clinical testing results, only basic demographic information, including age and sex, was available. Primary anatomic subsite and location of tissue specimens were provided by the treating physicians.

For analyses, patients were stratified into *PBRM1*-mut and *PBRM1*-wildtype (wt) cases. Only samples with pathogenic or presumed pathogenic mutations were classified as *PBRM1*-mut. Samples with benign or presumed benign *PBRM1* mutations or *PBRM1* variants of unknown significance were categorized as *PBRM1*-wt. Notably, germline testing was not performed as part of the tumor molecular testing.

### Immunohistochemistry (IHC)

IHC was performed on 1670 formalin-fixed paraffin-embedded (FFPE) tumor samples. Sections (4 µm) mounted on slides were stained using an automated system (Benchmark, Ventana Medical Systems, Tucson, Arizona, USA; Autostainer, DAKO, Carpinteria, California) according to the manufacturer’s instructions, and were optimized and validated according to CLIA/CAO and ISO requirements. All proteins of interest were evaluated on tumor cells. An intensity score (0 = no staining; 1+ = weak staining; 2+ = moderate staining; 3+ = strong staining) and a proportion score to determine the percentage of cells staining positive (0–100%) were used. The primary antibody used to detect PD-L1 expression was SP142 (Spring Biosciences, Pleasanton, CA, USA). The staining was considered positive if its intensity on the membrane of tumor cells was ≥2+ and the percentage of positively stained cells was ≥5. Analysis and scoring of IHC staining were performed by a board-certified pathologist.

### Microdissection

To increase the tumor density of samples undergoing DNA/RNA extraction for next-generation sequencing, microdissection was performed for all tumor samples, and the areas of the slides with the highest concentration of cancer cells were separated from the area of normal cells. The H&E slides were reviewed under a light microscope by a pathologist, or by a trained pathologist assistant. The tumor areas were marked and the non-tumor areas were avoided; the slides designated for microdissection were then stained with a nuclear fast red stain (NFR), and the tumor areas of the NFR stained slides were then manually dissected for DNA/RNA extraction.

### Next-generation sequencing (NGS)

During the recruitment period, tests have varied since technologies continuously evolved over time. The next-generation sequencing (NGS) platform for tumors tested in 2015 or earlier used the MiSeq platform (45 genes included; https://www.illumina.com/systems/sequencing-platforms/miseq.html), while those tested after 2015 were sequenced with the NextSeq platform (592 genes included; https://www.illumina.com/systems/sequencing-platforms/nextseq-1000-2000.html). NGS was performed on genomic DNA isolated from FFPE tumor samples using the NextSeq (*N* = 1292) or NovaSeq 6000 platforms (*N* = 577) (Illumina, Inc., San Diego, CA; https://www.illumina.com/systems/sequencing-platforms/novaseq.html). For NextSeq sequenced tumors, a custom-designed SureSelect XT assay was used to enrich 592 whole-gene targets (Agilent Technologies, Santa Clara, CA). For NovaSeq, more than 700 clinically relevant genes at high coverage and high read-depth were used, along with another panel designed to enrich for additional >20,000 genes at a lower depth. All variants were detected with >99% confidence based on allele frequency and amplicon coverage, with an average sequencing depth of coverage of >500 and an analytic sensitivity of 5%.

Prior to molecular testing, tumor enrichment was achieved by harvesting targeted tissue using manual microdissection techniques. Genetic variants identified were interpreted by board-certified molecular geneticists and categorized as ‘pathogenic,’ ‘likely pathogenic,’ ‘variant of unknown significance,’ ‘likely benign,’ or ‘benign,’ according to the American College of Medical Genetics and Genomics (ACMG) standards. When assessing mutation frequencies of individual genes,’ pathogenic,’ and ‘likely pathogenic’ were defined as mutations. The CAN of each exon was determined by calculating the average depth of the sample along with the sequencing depth of each exon and comparing this calculated result to a pre-calibrated value.

### Microsatellite instability (MSI)/DNA mismatch repair (MMR) status

A combination of multiple test platforms was used to determine the MSI or MMR status of the profiled samples, including fragment analysis (FA, Promega, Madison, WI), IHC (MLH1, M1 antibody; MSH2, G2191129 antibody; MSH6, 44 antibodies; and PMS2, EPR3947 antibody (Ventana Medical Systems, Inc., Tucson, AZ) and NGS (7000 target microsatellite loci were examined and compared to the reference genome hg19 from the University of California). The three platforms generated highly concordant results as previously reported^41^ and in the rare cases of discordant results, the MSI or MMR status of the tumor was determined in the order of IHC, FA, and NGS.

### Tumor mutational burden

Tumor mutational burden (TMB) was measured by counting all non-synonymous missense, nonsense, in-frame insertion/deletion, and frameshift mutations found per sample that had not been previously described as germline alterations in dbSNP151, Genome Aggregation Database (gnomAD) databases or benign variants identified by geneticists of Caris Life Sciences. A cutoff point of ≥10 mutations (mt) per megabase (MB) was used based on the KEYNOTE-158 pembrolizumab trial^42^, which showed that patients with a TMB of ≥10 mt/MB across several tumor types had higher response rates than patients with a TMB of <10 mt/MB. Caris Life Sciences is a participant in the Friends of Cancer Research TMB Harmonization Project^43^.

### Compliance statement

This study was conducted in accordance with the guidelines of the Declaration of Helsinki, the Belmont Report, and the U.S. Common Rule. In keeping with 45 CFR 46.101(b), this study was performed using retrospective, de-identified clinical data, and thus was considered IRB exempt from the ethical committee of the Medical University of Innsbruck. Written informed consent for the publication of clinical details was obtained from the presented patient. A copy of the consent form is available for review by the editor of this journal.

### Cell line

EGI1 cells (ACC-385) and CCC5 cells (ACC-810; Results provided as Supplementary Fig. S1) were obtained from the German Collection of Microorganisms and Cell Cultures GmbH (DSMZ, Braunschweig, Germany). Cells were cultured in 90% Modified Eagles Medium (MEM) plus 10% fetal calf serum, 1 mM sodium pyruvate, 4 mM l-glutamine, 1% penicillin–streptomycin and 2×MEM amino acids (both essential and non-essential), according to DSMZ recommendations. Short tandem repeat profiling was performed to ensure cell lineage identity. Cells were incubated at 37 °C with 5% CO_2_ and passaged every 3–4 days at confluency. DNA extraction and whole exome sequencing (Twist Comprehensive Exome) were accomplished by use of the NovSeq S1 (300 cycles) sequencing kit version 1.5. The primary analysis, secondary analysis, and annotation were performed with the Dragen Bio-IT pipeline.

### siRNA and transfection

Designed *PBRM1* siRNA-oligos were obtained from Integrated DNA Technologies (IDT, Coralville, Iowa, USA). We tested three different siRNAs designed to target PBRM1 (hs.Ri.PBRM1.13.1, 13.2, and 13.3, respectively), but only the hs.Ri.PBRM1.13.2 lead to a significant knockdown with a remaining gene expression of ~30% (data not shown), which we used for all further experiments. The sequences of the hs.Ri.PBRM1.13.2 siRNAs targeting *PBRM1* were 5′-GAAGAGGUUUUCACUCUCUGCUAAA-3′ and its complementary anti-sense 5′-UUUAGCAGAGAGUGAAAACCUCUUCAA-3′. To prove the high specificity of the siRNA oligos used and to avoid possible off-target effects, we performed an NCBI Megablast analysis using the human reference transcriptome and a BLASTn analysis using the human reference genome. Both analyses revealed high specificity to all transcript isoforms of the *PBRM1* gene. Although our study had the limitation of a single effective siRNA, we selected after bioinformatic prediction this siRNA due to its low probability to produce possible off-target effects on other genes involved in DNA remodeling and repair. A non-targeting siRNA (Sense: 5′-CCUUCCUCUCUUUCUCUCCCUUGUG-3′, Antisense: 3′-CACAAGGGAGAGAAAGAGAGGAAGG-5′) was used as control. Cells were reverse transfected using Lipofectamine RNAiMax (Invitrogen, Waltham, Massachusetts, USA), which was prepared according to the manufacturer’s instructions using OptiMEM reduced serum medium (Life Technologies, Carlsbad, CA, USA). The final good concentrations were 30 nM siRNA and 0.1 µL Lipofectamine RNAiMax. After transfection, cells were seeded in 96-well plates at 5000 cells/well-using medium without antibiotics and were allowed to rest for 24 h before treatment.

### Drug treatment and proliferation assay

The experimental setup has been described previously^11^. In brief, 24 h after seeding, cells were treated with medium and antibiotics containing 0.1, 0.5, 1, 5, 10, 25, or 50 µM Niraparib (Selleckchem, Houston, TX), 0.1, 0.5, 1, 5, 10, 50, 100, or 200 µM Olaparib (Selleckchem, Houston, TX), 0.01, 0.05, 0.1, 0.25, 0.5, 0.75, or 1 µM Berzosertib (Axon Medchem, Groningen, NL), or a combination of the respective concentrations of Berzosertib and Niraparib (all final well concentrations) and were incubated at 37 °C with 5% CO_2_ for six days. Medium containing the respective drug concentrations was replaced every 48 h. On day 6, 100 µM 5′-bromo-2′desoxyuridine (BrdU) labeling solution was added and the rate of proliferating cells was determined 24 h later (=d7) by means of a BrdU-ELISA (Roche, Basel, SUI), according to the manufacturer’s instructions. Optical density was measured at a wavelength of 370 nm and a reference wavelength of 492 nm using a microplate reader (Tecan Spark, Männedorf, SUI).

### mRNA quantification

At 24, 48, 72, 96, 120, 144, and 168 h after transfection, RNA was isolated from pelleted cells using Qiagen RNEasy Mini Kits (Hilden, GER). RT-qPCR was performed using Luna Universal OneStep RT-qPCR kits (New England Biolabs, Ipswich, USA) on a Quantstudio 3 (Thermo Fischer, Waltham, USA) according to the manufacturer’s instructions. RT-qPCR primers for *PBRM1* and *GAPDH* were obtained from Eurofins Genomics (Ebersberg, Germany; Sequence GAPDH-primer (Forward: 5′-CAA-TGA-CCC-CTT-CAT-TGA-CC-3′, and Reverse: 5′-TTG-ATT-TTG-GAG-GGA-TCT-GG-3′) and PBRM1-primer (Forward: 5′-AGG-AGG-AGA-CTT-TTC-AAT-CTT-CC-3′, and Reverse: 5′-CTT-CGC-TTT-GGT-GCC-CTA-ATG-3′). Relative gene expression was calculated by the ΔΔCT method^44,45^, using *GAPDH* expression levels to normalize *PBRM1* expression levels.

### Protein quantification

Protein extracts were obtained 24, 48, 72, 96, 120, 144, or 168 h after transfection using radioimmunoprecipitation assay buffer. Immunoblotting was performed using the Simple Western Jess platform (Bio-Techne, San Jose, CA). The final loading protein concentration was 1 µg/µL. For the detection of *PBRM1*, an anti-PBRM1 rabbit polyclonal antibody (A301-591A-M, Bethyl Laboratories, Montgomery) was used at a 1:100 dilution. GAPDH was used as a loading control (14C10 Rabbit mAB, Cell Signaling Technology, Danvers; 1:200 dilution). Uncropped images of the Jess Immunoblots are depicted in Supplementary Fig. 8 and Supplementary Fig. 9.

### Statistical analyses

The *χ*^2^ and two-sided Mann–Whitney *U* test were used to compare groups of the genomic profiling cohort. If applicable, we corrected *p*-values for multiple comparisons (*q*-value) using the Benjamini–Hochberg method. A *q*-value and a *p*-value < 0.05 were considered statistically significant. Least-squares, four-parameter, non-linear curve fitting, and calculation of LogIC_50_ values were performed using GraphPad Prism 9. Exploratory real-world overall survival was estimated from insurance claims and days to event were calculated from tissue collection to the last day of contact. We considered a patient deceased if the patient did not have an additional insurance record for 100 days (inferred death)- Kaplan–Meier estimates were calculated for molecularly defined patient cohorts. Significance was determined as *p*-value < 0.05.

### Reporting summary

Further information on research design is available in the Nature Research Reporting Summary linked to this article.

## Supplementary information

REPORTING SUMMARY

Supplementary Information

## Data Availability

The datasets generated and/or analyzed during the current study are available from the corresponding author on request. The de-identified sequencing data is owned by Caris Life Sciences, and cannot be publicly shared due to the data usage agreement signed by Dr. Andreas Seeber. However, qualified researchers can apply for access to these summarized data by contacting Joanne Xiu, Ph.D. (jxiu@carisls.com), and signing a data usage agreement. The processed NGS sequencing data are available at https://figshare.com/s/c47d6862584567cf6599.
