# Supplementary material for: PBRM1 mutations might render a subtype of biliary tract cancers sensitive to drugs targeting the DNA damage repair system
Source: NPJ Precis Oncol. 2023 Jul 3;7:64. doi: 10.1038/s41698-023-00409-5 (PMC10317977; doi:10.1038/s41698-023-00409-5)
Supplement: Supplementary file 2 — Supplementary Information [file 41698_2023_409_MOESM2_ESM.docx]

**Supplementary Information**


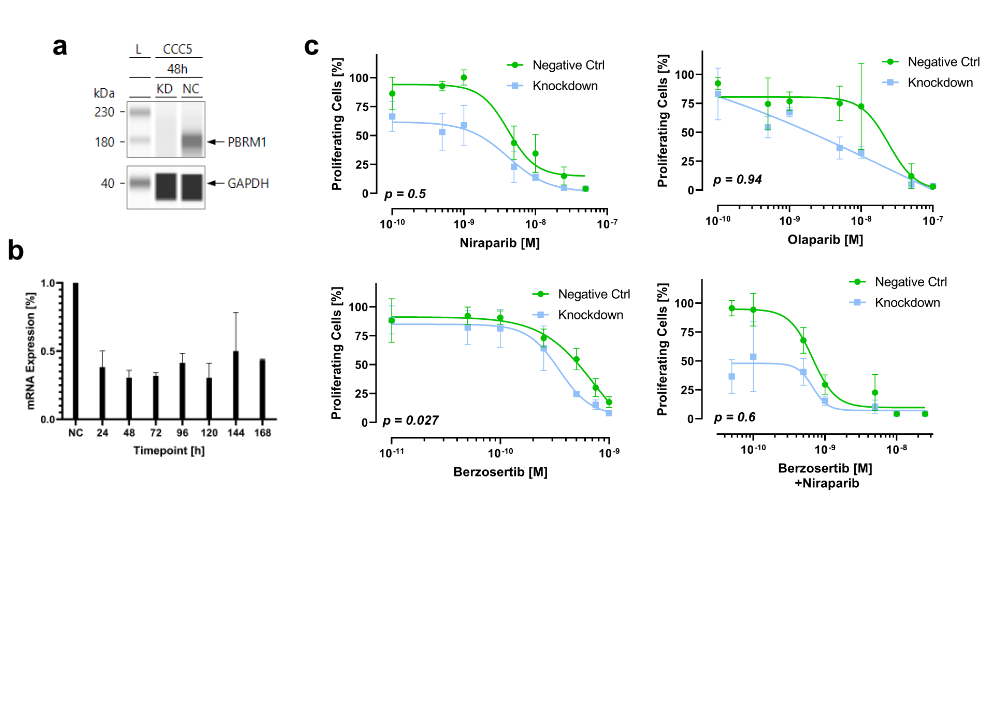


**Supplementary Figure 1:**

(a) Immunoblot showing successful siRNA mediated knockdown of PBRM1 after 48h in the CCC5 cell line. (b) Bar plot showing the remaining mRNA-expression level after siRNA knockdown of *PBRM1* in the CCC5 cell line over the course of drug treatment. (c) Dose-response curves of *PBRM1*-knockdown vs negative control in the CCC5 cell line treated with niraparib, olaparib, berzosertib and the combination of berzosertib and niraparib. Error-bars show mean and standard deviation.


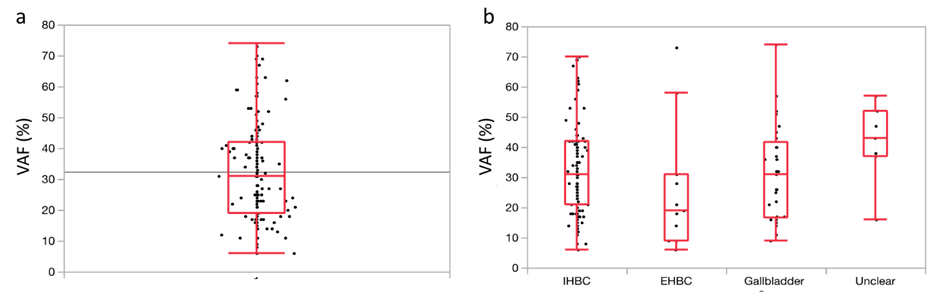


**Supplementary Figure 2:**

(a) Boxplot showing the distribution of Variant Allele Frequency (VAF) of pathogenic/likely pathogenic *PBRM1* mutations in the cohort (median 31%, mean 32.4%, range: 6-74%). (b) Distribution of VAF stratified by anatomic location (Intrahepatic (IHBC) median: 31%, mean: 32.8%, range: 6%-70%, extrahepatic (EHBC) median: 19%, mean: 26%, range: 6-73%, Gallbladder median 31%, mean 31%, range: 9-74%, unclear location median 43%, mean: 41.4%, range 16-57%). No significance in pairwise Mann-Whitney U test. (Boxplots elements definition: center line: median, box limits: upper and lower quartiles; whiskers: range; points: individual samples)


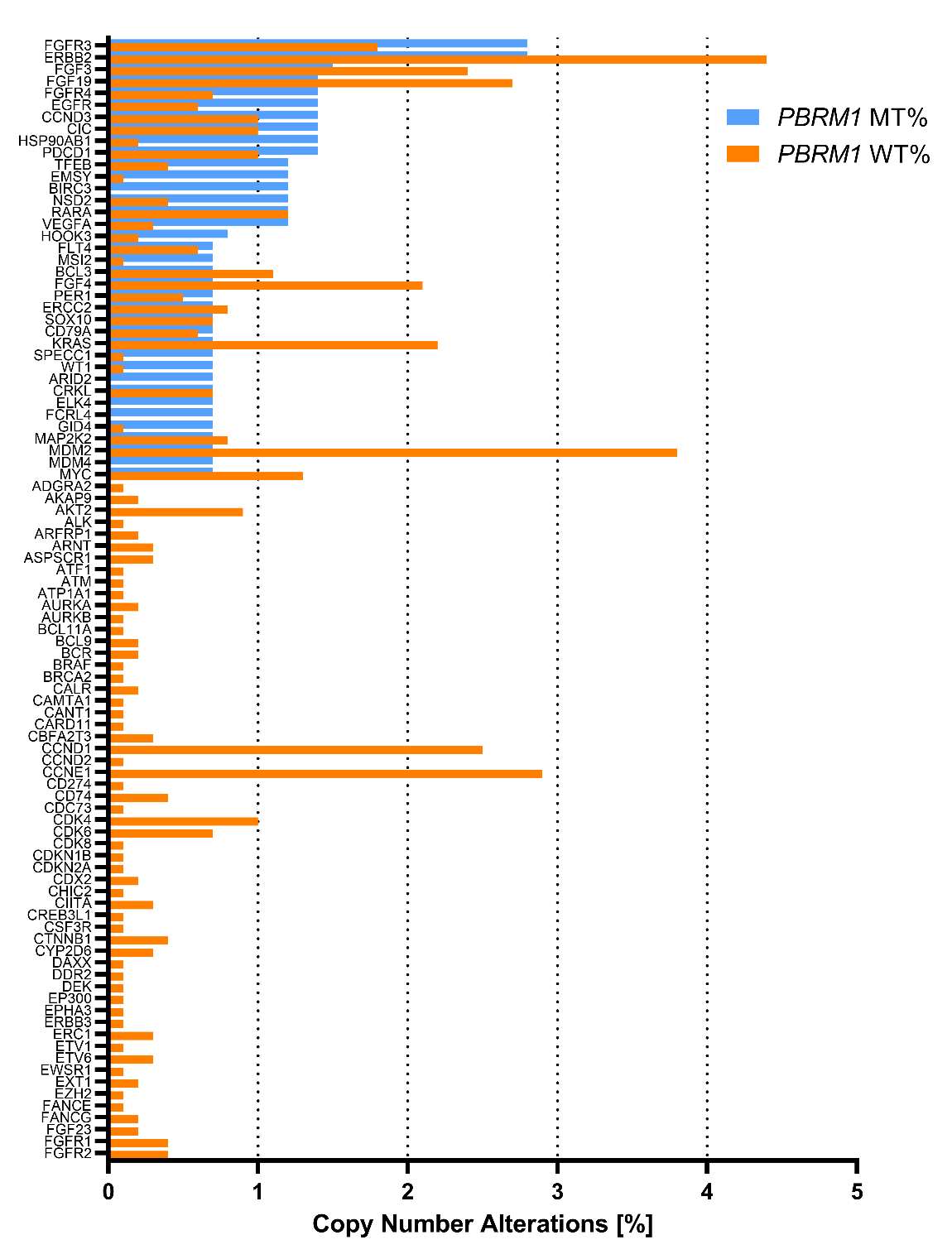


**Supplementary Figure 3:**

Rate of Copy Number Alterations (CNAs) identified in the study cohort.


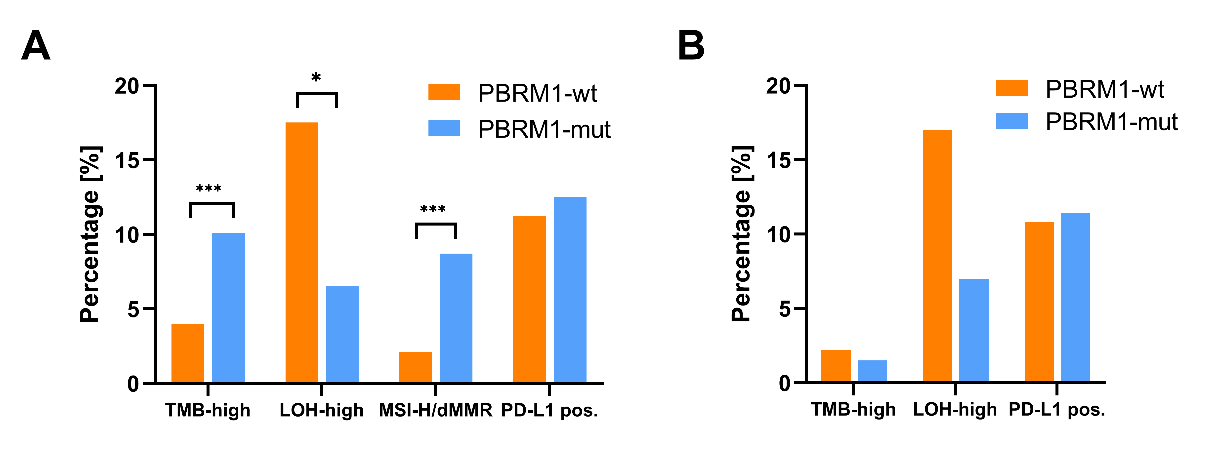


**Supplementary Figure 4:**

Comparison of TMB, LOH and PD1 status in both the overall cohort (A) and the MSS cohort (B) *p<0.05, **p<0.01. *p<0.001

***

***

**

**Supplementary Supplementary Figure 5:**

Comparison of TMB according to anatomic location. TMB was lower in intrahepatic tumors (median 3mut/mb, mean 5.8 mut/mb) compared to extrahepatic (median 4 mut/mb, mean 7.5 mut/mb p = 0.014)) and gallbladder (4mut/mb, mean = 10 mut/mb, p <0.0001). (Boxplots elements definition: center line: median, box limits: upper and lower quartiles; whiskers: range; points: individual samples). *p<0.05, **p<0.01. ***p<0.001


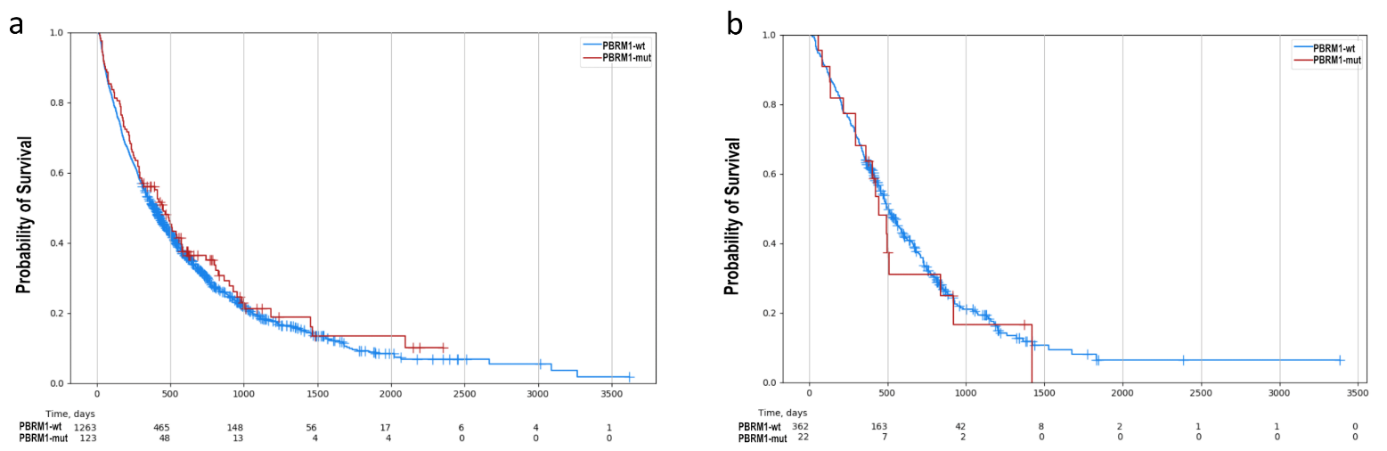


**Supplementary Figure 6:**

Kaplan-Meier Analysis for real world survival for (a) intrahepatic (Median days-to-event for *PBRM1*-wt: 388 days, for *PBRM1*-mut: 451 days. HR: 1.107, 95%CI 0.891-1.376,p=0.358) and (b) extrahepatic BTCs (Median days-to-event for *PBRM1*-wt: 495 days, for *PBRM1*-mut: 492 days. HR: 0.933, 95%CI 0.601-1.448, p=0.358) stratified by *PBRM1*-mutation status.


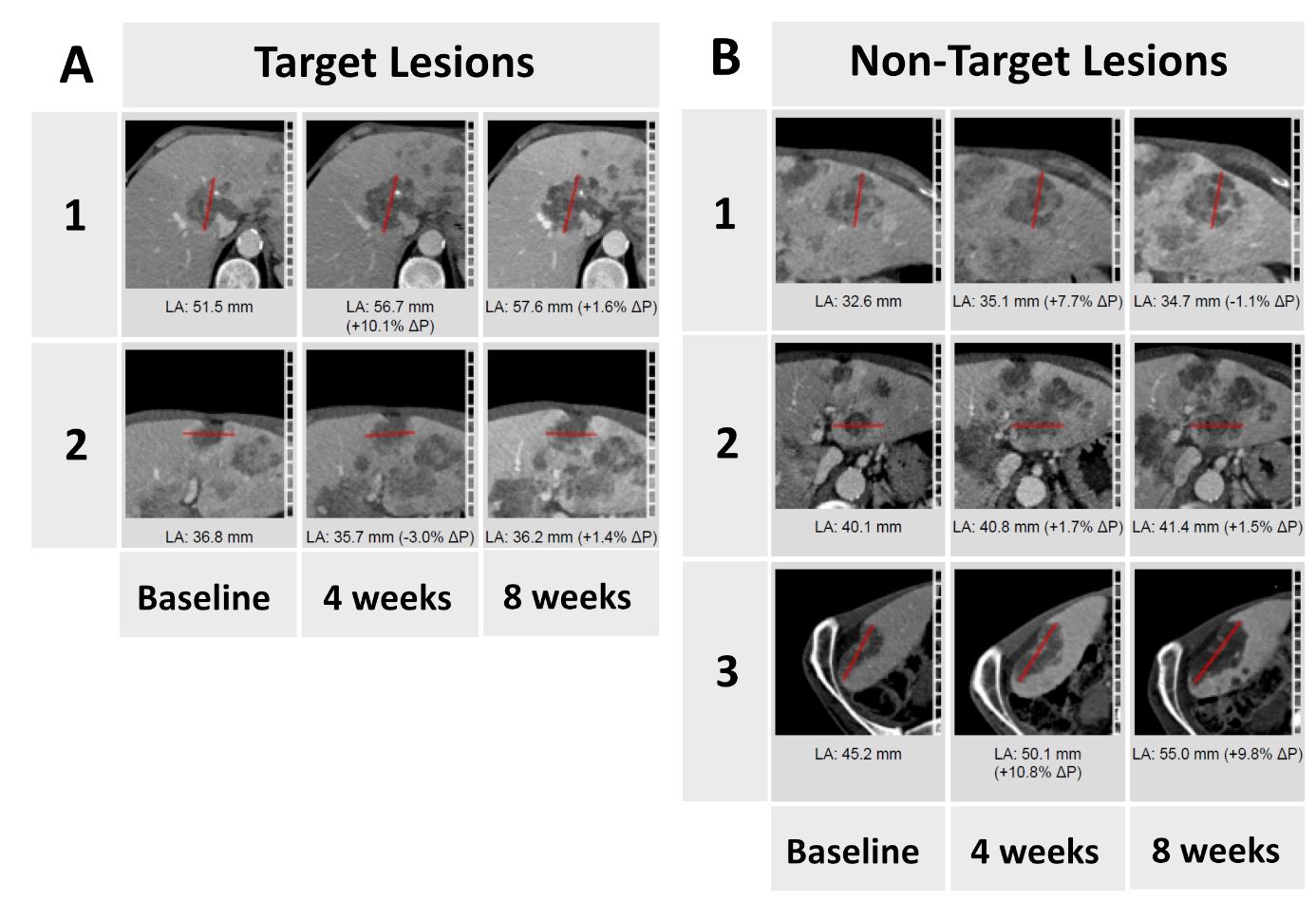


**Supplementary Figure 7:**

Radiologic evaluation of (A) target and (B) non-target lesions of the reported patient according to RECIST v1.1 showing heterogenous dynamics in response to niraparib treatment with SD after four and eight weeks.


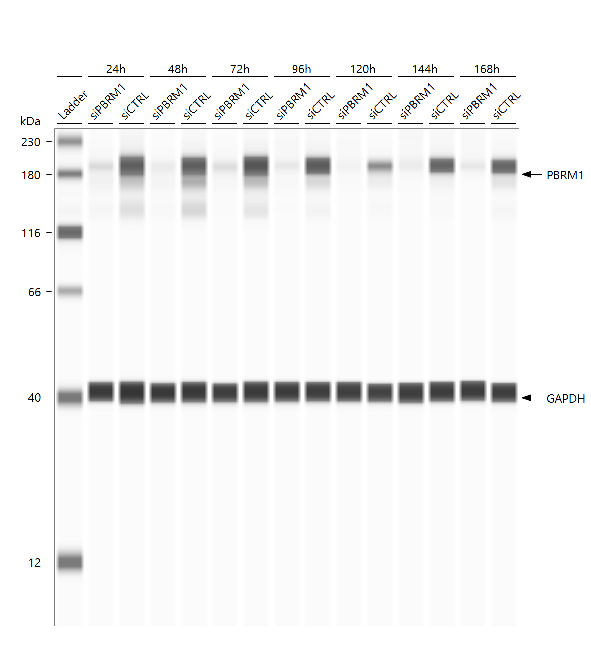


**Supplementary Figure 8:**

Uncropped JESS SimpleWestern Immunoblot of siPBRM1 treated EGI-1 cells (see also Figure 3a)


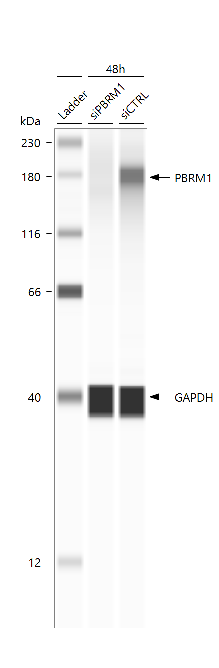


**Supplementary Figure 9:**

Uncropped JESS SimpleWestern Immunoblot of siPBRM1 treated CCC5 cells (see also Supplementary Figure 1)

**Supplementary Table 1:**

List of all pathogenic or likely pathogenic variants in *PBRM1* identified in the study cohort.

| **CDS** | **AA-Change** | **Result** |
| --- | --- | --- |
| c.3765dupT | A1256fs | Pathogenic Variant |
| c.4110dupA | A1371fs | Pathogenic Variant |
| c.4530_4536del7 | A1512fs | Pathogenic Variant |
| c.1087+1G>A | N.A. | Pathogenic Variant |
| c.1087+1G>T | N.A. | Pathogenic Variant |
| c.1088-54_1099del66 | N.A. | Pathogenic Variant |
| c.1444-1G>A | N.A. | Pathogenic Variant |
| c.1924+1G>A | N.A. | Pathogenic Variant |
| c.237-1G>T | N.A. | Pathogenic Variant |
| c.2949_2965+10del27 | N.A. | Pathogenic Variant |
| c.2965+1G>C | N.A. | Pathogenic Variant |
| c.2965+1G>T | N.A. | Pathogenic Variant |
| c.3048+1G>T | N.A. | Pathogenic Variant |
| c.3049-2A>T | N.A. | Pathogenic Variant |
| c.3312+2T>C | N.A. | Pathogenic Variant |
| c.3313-2A>T | N.A. | Pathogenic Variant |
| c.3459-1G>A | N.A. | Pathogenic Variant |
| c.3459-1G>T | N.A. | Pathogenic Variant |
| c.3459-1G>T | N.A. | Pathogenic Variant |
| c.3459-1G>T | N.A. | Pathogenic Variant |
| c.3617-2A>C | N.A. | Pathogenic Variant |
| c.3617-3_3622del9 | N.A. | Pathogenic Variant |
| c.4360-2A>T | N.A. | Pathogenic Variant |
| c.4577-13_4581del18 | N.A. | Pathogenic Variant |
| c.645+1G>C | N.A. | Pathogenic Variant |
| c.813+1G>T | N.A. | Pathogenic Variant |
| c.996-1G>T | N.A. | Pathogenic Variant |
| c.996-1G>T | N.A. | Pathogenic Variant |
| c.204_205delCT | C69fs | Pathogenic Variant |
| c.204_205delCT | C69fs | Pathogenic Variant |
| c.471dupA | D158fs | Pathogenic Variant |
| c.51_56delinsA | D18fs | Pathogenic Variant |
| c.1658dupA | D554fs | Pathogenic Variant |
| c.3318dupA | E1107fs | Pathogenic Variant |
| c.3564dupA | E1189fs | Pathogenic Variant |
| c.3564dupA | E1189fs | Pathogenic Variant |
| c.3821dupA | E1275fs | Pathogenic Variant |
| c.3877G>T | E1293* | Pathogenic Variant |
| c.4018G>T | E1340* | Pathogenic Variant |
| c.4500dupA | E1501fs | Pathogenic Variant |
| c.523G>T | E175* | Pathogenic Variant |
| c.676G>T | E226* | Pathogenic Variant |
| c.1060_1061delGA | E354fs | Pathogenic Variant |
| c.1480G>T | E494* | Pathogenic Variant |
| c.1602_1618del17 | E535fs | Pathogenic Variant |
| c.1714G>T | E572* | Pathogenic Variant |
| c.1807delG | E603fs | Pathogenic Variant |
| c.2386G>T | E796* | Pathogenic Variant |
| c.2536G>T | E846* | Pathogenic Variant |
| c.256G>T | E86* | Pathogenic Variant |
| c.256G>T | E86* | Pathogenic Variant |
| c.2599G>T | E867* | Pathogenic Variant |
| c.255dupT | E86fs | Pathogenic Variant |
| c.2722_2723insT | E908fs | Pathogenic Variant |
| c.2971G>T | E991* | Pathogenic Variant |
| c.2971G>T | E991* | Pathogenic Variant |
| c.2970delA | E991fs | Pathogenic Variant |
| c.57delT | F19fs | Pathogenic Variant |
| c.213_214insA | F72fs | Pathogenic Variant |
| c.2616delT | F872fs | Pathogenic Variant |
| c.2977_2989del13 | F993fs | Pathogenic Variant |
| c.66delG | H23fs | Pathogenic Variant |
| c.835delA | I279fs | Pathogenic Variant |
| c.835dupA | I279fs | Pathogenic Variant |
| c.2940delT | I980fs | Pathogenic Variant |
| c.3056_3057dupTT | K1020fs | Pathogenic Variant |
| c.3057dupT | K1020fs | Pathogenic Variant |
| c.3846delG | K1283fs | Pathogenic Variant |
| c.630_631delAC | K210fs | Pathogenic Variant |
| c.1248_1276del29 | K416fs | Pathogenic Variant |
| c.1311_1332del22 | K438fs | Pathogenic Variant |
| c.1453A>T | K485* | Pathogenic Variant |
| c.1678A>T | K560* | Pathogenic Variant |
| c.180dupT | K61fs | Pathogenic Variant |
| c.1953delA | K651fs | Likely Pathogenic Variant |
| c.2009_2024del16 | K670fs | Pathogenic Variant |
| c.2105delA | K702fs | Pathogenic Variant |
| c.2719_2720delAA | K907fs | Pathogenic Variant |
| c.3881T>A | L1294* | Pathogenic Variant |
| c.4492delC | L1498fs | Pathogenic Variant |
| c.2654_2655dupTT | L886fs | Pathogenic Variant |
| c.2727dupA | L910fs | Pathogenic Variant |
| c.773delA | N258fs | Pathogenic Variant |
| c.773delA | N258fs | Pathogenic Variant |
| c.773delA | N258fs | Pathogenic Variant |
| c.773dupA | N258fs | Pathogenic Variant |
| c.773dupA | N258fs | Pathogenic Variant |
| c.773dupA | N258fs | Pathogenic Variant |
| c.773dupA | N258fs | Pathogenic Variant |
| c.773dupA | N258fs | Pathogenic Variant |
| c.773dupA | N258fs | Pathogenic Variant |
| c.773dupA | N258fs | Pathogenic Variant |
| c.998_1029del32 | N333fs | Pathogenic Variant |
| c.2645dupA | N882fs | Pathogenic Variant |
| c.2645dupA | N882fs | Pathogenic Variant |
| c.32delC | P11fs | Pathogenic Variant |
| c.3815delC | P1272fs | Pathogenic Variant |
| c.3826_3827delCC | P1276fs | Pathogenic Variant |
| c.4313delC | P1438fs | Pathogenic Variant |
| c.4430_4431delCT | P1477fs | Pathogenic Variant |
| c.349C>T | Q117* | Pathogenic Variant |
| c.3817C>T | Q1273* | Pathogenic Variant |
| c.3817C>T | Q1273* | Pathogenic Variant |
| c.563_575del13 | Q188fs | Pathogenic Variant |
| c.1291C>T | Q431* | Pathogenic Variant |
| c.1429C>T | Q477* | Pathogenic Variant |
| c.1432C>T | Q478* | Pathogenic Variant |
| c.1816C>T | Q606* | Pathogenic Variant |
| c.2609_2637del29 | Q870fs | Pathogenic Variant |
| c.267_268delinsA | Q90fs | Pathogenic Variant |
| c.3079C>T | R1027* | Pathogenic Variant |
| c.3079C>T | R1027* | Pathogenic Variant |
| c.3079C>T | R1027* | Pathogenic Variant |
| c.3185dupC | R1063fs | Pathogenic Variant |
| c.3478C>T | R1160* | Pathogenic Variant |
| c.3478C>T | R1160* | Pathogenic Variant |
| c.1300delC | R434fs | Pathogenic Variant |
| c.1564C>T | R522* | Pathogenic Variant |
| c.1600C>T | R534* | Pathogenic Variant |
| c.2128C>T | R710* | Pathogenic Variant |
| c.2128C>T | R710* | Pathogenic Variant |
| c.2548C>T | R850* | Pathogenic Variant |
| c.2548C>T | R850* | Pathogenic Variant |
| c.2548C>T | R850* | Pathogenic Variant |
| c.2548C>T | R850* | Pathogenic Variant |
| c.2626C>T | R876C | Likely Pathogenic Variant |
| c.2761C>T | R921* | Pathogenic Variant |
| c.2761C>T | R921* | Pathogenic Variant |
| c.2776delA | R926fs | Pathogenic Variant |
| c.36_48del13 | S13fs | Pathogenic Variant |
| c.1028C>G | S343* | Pathogenic Variant |
| c.1028C>G | S343* | Pathogenic Variant |
| c.1028C>G | S343* | Pathogenic Variant |
| c.1400C>G | S467* | Pathogenic Variant |
| c.1540delA | S514fs | Pathogenic Variant |
| c.1953dupA | S652fs | Pathogenic Variant |
| c.1953dupA | S652fs | Pathogenic Variant |
| c.1953dupA | S652fs | Pathogenic Variant |
| c.2363C>A | S788* | Pathogenic Variant |
| c.4086delG | T1363fs | Pathogenic Variant |
| c.3043_3044delGT | V1015fs | Pathogenic Variant |
| c.1434dupA | V479fs | Pathogenic Variant |
| c.3792C>G | Y1264* | Pathogenic Variant |
| c.4404T>A | Y1468* | Pathogenic Variant |
| c.543C>A | Y181* | Pathogenic Variant |
| c.1225delT | Y409fs | Pathogenic Variant |
| c.1248dupA | Y417fs | Pathogenic Variant |
| c.1407delC | Y470fs | Pathogenic Variant |
| c.1665T>G | Y555* | Pathogenic Variant |
| c.2400C>A | Y800* | Pathogenic Variant |
| c.251delA | Y84fs | Pathogenic Variant |

**Supplementary Table 2:**

Selected results of exome sequencing of the EGI1 and CCC5 cell line:

| **Cell Line** | **Gene** | **CDS** | **AA Change** | **Predicted**  **Consequence** | **Interpretation according to ClinVar (NCBI)** |
| --- | --- | --- | --- | --- | --- |
| EGI1 | KRAS | c.35G>A | p.(Gly12Asp) | Missense | Pathogenic |
|  | TP53 | c.818G>A | p.(Arg273His) | Missense | Pathogenic |
|  | BRCA1 | c.1067A>G | p.(Gln356Arg) | Missense | Benign |
|  | ATM | c.170G>A | p.(Trp57Ter) | Nonsense | Pathogenic |
|  | MSH3 | c.2830_2831dupAA | p.(Asn944LysfsTer14) | Frameshift | unknown |
| CCC5 | KRAS | c.35G>A | p.(Gly12Asp) | Missense | Pathogenic |
|  | ARID1A | c.1656dupA | p.(Gln553ThrfsTer70) | Frameshift | unknown |
|  | TP53 | c.215C>G | p.(Pro72Arg) | Missense | Benign |
|  | PARP1 | c.2285T>C | p.(Val762Ala) | Missense | Benign |
|  | BRCA2 | c.1813delA | p.(Ile605TyrfsTer9) | Frameshift | Pathogenic |
|  | ATM | c.5948A>G | p.(Ser1983=) | Synonymous | Benign |
|  | ATR | c.7875G>A | p.(Gln2625=) | Synonymous | Benign |

**Supplementary Table 3:**

Radiologic evaluation of hepatic lesions of the presented patient according to RECIST v1.1 (see also Supplementary Figure S6).

| **LESION** | **Baseline** | **4-weeks FU** | **8-weeks FU** |
| --- | --- | --- | --- |
| Target Lesion 1  Segment VIII | 51.5mm | 56.7mm | 57.6mm |
| Target Lesion 2  Segment IVa | 36.8mm | 35.7mm | 36.2mm |
| non-Target Lesion 1  Segment II | 32.6mm | 35.1mm | 34.7mm |
| non-Target Lesion 1  Segment III | 40.1mm | 40.8mm | 41.4mm |
| non-Target Lesion 1  Segment VI | 45.2mm | 50.1mm | 55.0mm |
| Target Sum | 88.3mm | 92.4mm (+4.6% Baseline) | 93.8mm  (+6.2% Baseline/+1.5% to 4-weeks) |
| Target Response |  | Stable Disease | Stable Disease |
| Non-Target Response |  | Non-CR/Non-PD | Non-CR/Non-PD |
| Timepoint Response |  | Stable Disease | Stable Disease |
